# Selenium-Containing Organic Fertilizer Application Affects Yield, Quality, and Distribution of Selenium in Wheat

**DOI:** 10.3390/life13091849

**Published:** 2023-08-31

**Authors:** Peng Chen, Hiba Shaghaleh, Yousef Alhaj Hamoud, Jing Wang, Wenxia Pei, Xianfu Yuan, Jianjian Liu, Cece Qiao, Wenhui Xia, Jianfei Wang

**Affiliations:** 1 Key Lab of Bio-Organic Fertilizer Creation, Ministry of Agriculture and Rural Affairs, Anhui Science and Technology University, Bengbu 233030, China; chenpeng@ahstu.edu.cn (P.C.); chenpeng1631998@163.com (J.W.); peiwx@ahstu.edu.cn (W.P.); yuanxf@ahstu.edu.cn (X.Y.); liujj@ahstu.edu.cn (J.L.); qiaocece@ahstu.edu.cn (C.Q.); cp1551330423@163.com (W.X.); 2 College of Environment, Hohai University, Nanjing 210098, China; hiba-shaghaleh@hotmail.com; 3 College of Hydrology and Water Resources, Hohai University, Nanjing 210098, China; yousef-hamoud11@hotmail.com

**Keywords:** selenium, wheat yield, mineral nutrient, heavy metal

## Abstract

This study was designed to investigate the effect on wheat yield of applying organic fertilizers (OF) with five different selenium (Se) concentrations. The mineral nutrients, cadmium (Cd) content, and the distribution of Se in wheat plants were also measured. The results showed that wheat yields reached a maximum of 9979.78 kg ha^−1^ in Mengcheng (MC) County and 8868.97 kg ha^−1^ in Dingyuan (DY) County, Anhui Province, China when the application amount of selenium-containing organic fertilizer (SOF) was up to 600 kg ha^−1^. Among the six mineral nutrients measured, only the calcium (Ca) content of the grains significantly increased with an increase in the application amount of SOF in the two regions under study. Cd content showed antagonistic effects with the Se content of wheat grains, and when the SOF was applied at 1200 kg ha^−1^, the Cd content of the grains was significantly reduced by 30.1% in MC and 67.3% in DY, compared with under the Se0 treatment. After application of SOF, the Se content of different parts of the wheat plant ranked root > grain > spike-stalk > glume > leaf > stem. In summary, SOF application at a suitable concentration could increase wheat yields and significantly promote the Ca content of the grains. Meanwhile, the addition of Se effectively inhibited the level of toxic Cd in the wheat grains.

## 1. Introduction

Selenium (Se) is a beneficial element for all plants, and Se fertilization can promote plant growth and increase crop resistance to oxidative stress [1]. Se is referred to as the “monarch of anti-cancer” and “the spark of life” and is one of the trace elements that are necessary for human and animal survival. It can not only boost the human immune system but also has a variety of positive effects such as detoxification by combining with heavy metals in the body [2,3,4,5]. Studies have revealed that Se may be associated with more than 40 diseases in humans and animals and that Se deficiency is not only a major cause of Creutzfeldt–Jakob disease and macrosomia [6,7] but also increases the risk of cancer, cataracts, and many other diseases [8,9]. However, the distribution of Se in the earth’s crust is extremely uneven, resulting in a severe Se deficiency in the soil of most countries and regions [10]. China is one of the countries with the most severe Se deficiency in the world, and 72% of China’s area is Se deficient [11]. It can be seen that the difference in the geographical distribution of Se has led to the deficiency of Se in the human body in many regions. Meanwhile, ensuring that the body receives a proper amount of Se has also become a research hotspot. However, excessive Se supplementation is not only ineffective but can also cause Se toxicity, which can result in diabetes and a host of other conditions [12,13]. Consequently, proper Se supplementation is gaining more and more attention. Various nations have established different standards for recommended Se consumption, which are based on the various Se contents in regional foods and the actual Se intake for residents. There are different ways to supplement the body’s Se, but diet and medication are the two primary ways to deliver Se element. According to research, taking Se element from food is the most secure and efficient approach currently [14].

In nature, Se is mainly bound to plant proteins in the form of selenomethionine (SeMet) and selenocysteine (SeCys) [15]. Plants, as intermediary carriers of Se flow in the soil–plant–human system, play a crucial role in regulating Se levels in the human body [16]. Selenium can have both a positive effect and cause serious negative effects on plants, depending on the concentration of Se and the type of plant [17]. The effects of various abiotic stresses on plants can be mitigated by the appropriate concentration of selenium [18,19]. However, the distribution of Se in the soil is extremely uneven. Only a very few areas in China are Se-rich, so it is not feasible to solve the problem of Se deficiency with natural Se-rich crops for millions of people [20]. As a result, currently, the primary method for enriching levels of Se in crops is through exogenous delivery of Se. Leaf spraying and soil application are the primary methods of exogenous Se application.

In most developing countries, wheat is the main food source. Foods made from wheat provide more than 70% of daily calorie needs in China. To promote Se intake in the body, it is essential to increase the Se content in wheat grains [21]. In previous studies, exogenous Se has been proven to have a considerable influence on wheat metabolism, stress resistance, respiration, and photosynthesis, resulting in higher yield and enhanced wheat quality [22,23,24]. Cadmium (Cd), as the primary soil contaminant in China, has stronger chemical activity and bioavailability in soil compared with other heavy metals. It can transfer to edible parts of crops via metabolic processes, then accumulate in the human body through transport through the food chain. An excess intake of Cd can cause bone diseases, cancer, and other diseases [25,26,27]. Consequently, it is critical to address the issue of soil Cd pollution. Studies have found that the presence of Se lowers Cd levels drastically in many crops, including rice and wheat [28,29]. This is because Se and heavy metals have a significant affinity for one another. As a result, when Se and Cd ions combine to form metal–Se–protein complexes in crops, the amount of free Cd can be lowered [30].

In this study, different rates of selenium-containing organic fertilizer (SOF) were applicated in wheat fields, then the effects of different Se concentrations on the wheat yield, the content of calcium (Ca), magnesium (Mg), iron (Fe), manganese (Mn), copper (Cu), zinc (Zn), and Cd in the grains, and the distribution of Se in wheat grains, glumes, spike-stalks, leaves, stems, and roots were investigated. The goal was to verify the proper concentration of SOF and analyze the inhibiting effect of the fertilizer on the levels of important elements in wheat. This study can provide theoretical references for the high-quality and efficient production of Se-rich wheat.

## 2. Materials and Methods

### 2.1. Experimental Design

This experiment was conducted from October 2021 to June 2022 in Mengcheng (MC, 32°58′28″ N, 116°35′57″ E) and Dingyuan (DY, 32°32′10″ N, 117°25′42″ E) Counties, Anhui Province, China. MC is a warm temperate semi-humid monsoon climate area, with an average annual temperature of 14.8 °C and average annual precipitation of 732.63 mm. DY is a transitional region from northern subtropical to a warm temperate climate, with an annual average temperature of 14.8 °C and annual average precipitation of 940.00 mm. The chemical properties of surface soils (0–15 cm) in the test areas are shown in Table 1. The wheat varieties used in the field trial in MC and DY were Huaimai 33 (spring wheat) and Yangmai 25 (semi-winter wheat), respectively. The OF and SOF were both bought from Fuyang Yifeng Fertilizer Co., Ltd., Fuyang, China. The OF was made by composting chicken manure and rice straw (chicken manure:rice straw = 7:3), while the SOF was made by adding sodium selenite during the composting process of the OF. The Se content of the SOF was 256.96 mg kg^−1^. By applying SOF and organic fertilizers (OF), the experiment designed five treatments with different concentrations of SOF (Se0, Se1, Se2, Se3, Se4), and the total fertilizer application was 1200 kg ha^−1^ in each treatment. No treatments were duplicated, and each treatment had an area of 5 m^2^. The specific fertilizer application ratios are shown in Table 2. In all treatments, the fertilizers were applied before wheat planting. Later field management was in line with local farmers’ usual practices.

### 2.2. Sample Collection and Measurement of Grain Yield

Three 1 m^2^ samples of wheat plants were randomly selected in each treatment at the wheat maturity stage, and all wheat plants of each plot in all treatments were harvested and placed in net bags. The number of spikes and kernels per spike, and the thousand kernel weight of wheat in each sample were then measured separately and the yield of grains was calculated.

### 2.3. Sample Preparation

The collected wheat samples were washed with deionized water and divided into six parts: grains, glumes, spike-stalks, leaves, stems, and roots. Each part of the wheat samples was dried in the oven (DHG-9070A, Shanghai, China) at 70 °C until the weight was stable. Then, the dried samples were ground into powders, and all the powders were sifted with a sieve of 0.25 mm. Finally, the obtained samples were stored in a plastic bag for the determination of Se, Cd, Ca, Mg, Fe, Mn, Cu, and Zn.

### 2.4. Determination of Total Se Content

A total of 0.1000 g of each sieved sample was put into a microwave digestion vessel and 10 mL of mixed acid solution (HNO_3_:H_2_O_2_ = 7:3, *v*/*v*) was added. All reagents are purchased from Sinopharm Chemical Reagent Co., Ltd. (Beijing, China), with guaranteed concentrations. The digestion vessels were placed in a microwave digestion system (JUPITER-B, Shanghai, China), and the digestion program was set to 150 °C for 10 min, 170 °C for 10 min, and 190 °C for 15 min. When the digestion process was finished, the digestion vessels were removed and placed on an acid-driven processor (TK12, Shanghai, China), and the temperature was set to 120 °C. A 5 mL aliquot of HCl (6 mol L^−1^) was added into the digestion vessels when the liquid volume left in the digestion vessels was less than 1 mL, then the solution was evaporated until it was clear and colorless. The solution was then diluted to 25 mL with ultrapure water and 10 mL of the mixed solution was transferred into a 15 mL centrifuge tube to which was added 1 mL of HCl (12 mol L^−1^) and 2 mL of potassium ferricyanide (100 g L^−1^). Finally, the total Se content in each part of the wheat samples was determined using a hydride atomic fluorescence spectrometer (PF52, Beijing, China).

### 2.5. Determination of Cd, Ca, Mg, Fe, Mn, Cu, and Zn Contents

The sample weight of wheat grains and amounts of added acid for the mineral element assays were the same as those for the determination of Se. After the samples were digested, the digestion vessels were put into an acid-driven processor (TK12, Shanghai, China) and the temperature was set to 160 °C. The digestion vessels were removed when the liquid volume left was less than 1 mL and the digestion solution was diluted to 50 mL with ultrapure water. The Cd, Ca, Mg, Fe, Mn, Cu, and Zn Contents of wheat grains were subsequently determined with an atomic absorption spectrometer (ZEEnit 700P, Jena, Germany).

### 2.6. Statistical Analyses

All statistical analyses were performed using Microsoft Excel 2021 and SPSS 26.0 software (IBM, Armonk, NY, USA), and Origin 2022 (OriginLab, Northampton, MA, USA) was used to plot the graphs. One-way ANOVA analysis was carried out to analyze between-group variance (*p* < 0.05). Error bars indicated standard deviation (SD). The data were then fitted into linear y = ax + b models to analyze the correlations.

## 3. Results

### 3.1. Wheat Grain Yield

**Figure 1:**
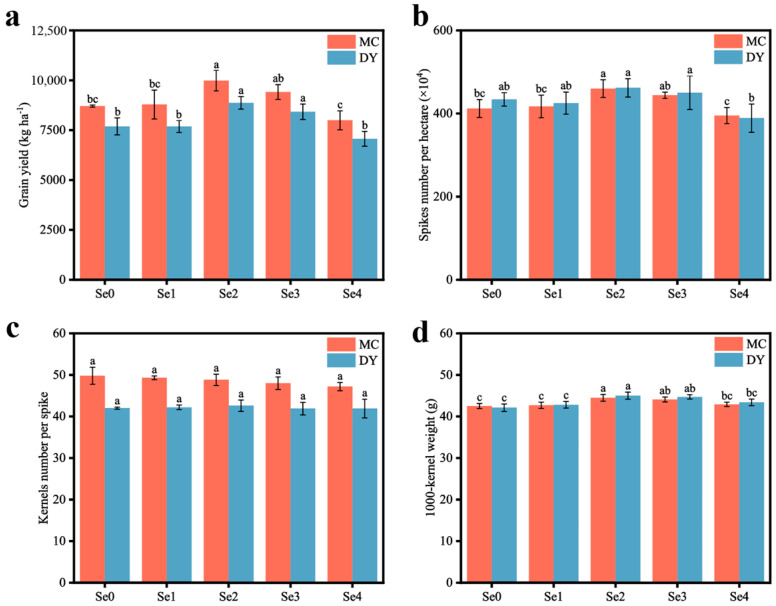
Grain yield (**a**), spike number per hectare (**b**), kernel number per spike (**c**), and 1000-kernel weight (**d**) of wheat under different ratios of selenium-containing organic fertilizers (SOF) and organic fertilizers (OF) in Mengcheng (MC) and Dingyuan (DY). Different lowercase letters indicate significant differences (*p* < 0.05) among different treatments using one-way ANOVA analysis with Duncan’s multiple range test and error bars show the ± standard deviation (SD). Se0: 0 kg ha^−1^ of SOF and 1200 kg ha^−1^ of OF. Se1: 300 kg ha^−1^ of SOF and 900 kg ha^−1^ of OF. Se2: 600 kg ha^−1^ of SOF and 600 kg ha^−1^ of OF. Se3: 900 kg ha^−1^ of SOF and 300 kg ha^−1^ of OF. Se4: 1200 kg ha^−1^ of SOF and 0 kg ha^−1^ of OF.

With an increase in SOF application, the yield of wheat at the MC and DY experimental sites first increased and then decreased, and reached the highest values at the two sites of 9979.78 kg ha^−1^ and 8868.97 kg ha^−1^, respectively, under the Se2 treatment. Compared with under the Se0 treatment, the wheat yield of MC and DY under the Se2 treatment significantly increased by 14.6% and 15.3%, while the wheat yield under the Se4 treatment significantly decreased by 8.18% and 8.09%, respectively (Figure 1a). The number of wheat spikes and 1000-kernel weight had a significant impact on the change in wheat yield (Figure 1b,d). Compared to the Se0 treatment, the Se2 treatment considerably enhanced the number of spikes per hectare and the 1000-kernel weight of wheat in the two regions. The increase in the number of spikes per hectare reached 11.7% and 6.5%, while the 1000-kernel weight increased by 4.7% and 6.9%, respectively. Conversely, there was no significant variation in kernel number per spike among the treatments.

### 3.2. The Content of Mineral Nutrients in Wheat Grains

**Figure 2:**
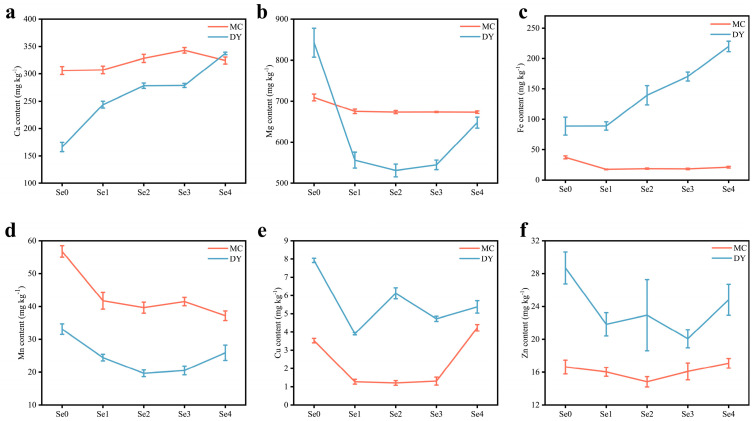
Calcium (Ca) content (**a**), magnesium (Mg) content (**b**), iron (Fe) content (**c**), manganese (Mn) content (**d**), copper (Cu) content (**e**), and zinc (Zn) content (**f**) of wheat grains under different ratios of selenium-containing organic fertilizers (SOF) and organic fertilizers (OF) in Mengcheng (MC) and Dingyuan (DY). One-way ANOVA analysis with Duncan’s multiple range test was used and error bars show the ± standard deviation (SD). Se0: 0 kg ha^−1^ of SOF and 1200 kg ha^−1^ of OF. Se1: 300 kg ha^−1^ of SOF and 900 kg ha^−1^ of OF. Se2: 600 kg ha^−1^ of SOF and 600 kg ha^−1^ of OF. Se3: 900 kg ha^−1^ of SOF and 300 kg ha^−1^ of OF. Se4: 1200 kg ha^−1^ of SOF and 0 kg ha^−1^ of OF.

Ca and Mg, two essential major elements for plants, responded differently to changes in Se content (Figure 2a,b). Ca content in the grains of the two regions increased significantly with the increasing application of SOF. The maximum Ca content in the grains was 342.98 mg kg^−1^ under the Se3 treatment in MC and 337.31 mg kg^−1^ under the Se4 treatment in DY. Compared to under the Se0 treatment, Ca content increased by 12.1% in MC and 102.9% in DY. The Mg content reached the minimum values of 672.99 mg kg^−1^ under the Se4 treatment in MC and 531.33 mg kg^−1^ under the Se2 treatment in DY. The reduction reached 5.1% in MC and 36.9% in DY compared to under the Se0 treatment. The Fe content in the grains of the two regions did not show a consistent trend (Figure 2c). Under SOF treatment, the Fe content of the grains from MC was lower than that under the Se0 treatment, dropping by 43.5% to 52.9%. However, the Fe content of the grains from DY increased significantly with SOF treatment compared to under the Se0 treatment, peaking at a 147.5% increase and 219.69 mg kg^−1^ under the Se4 treatment. The contents of Mn, Cu, and Zn all showed irregular trends with the increasing application of SOF (Figure 2d–f). Compared with the Se0 treatment, the reduction in Mn content of the grains ranged from 26.5% to 34.5% in MC and 21.7% to 40.6% in DY. The Cu content of the grains under the Se1 to Se3 treatments in MC was dramatically reduced by 62.9% to 65.7% and significantly increased by 19.8% under the Se4 treatment compared to the Se0 treatment. Under the Se1 treatment, the Cu content in the grains from DY was significantly decreased by 50.6% compared with under the Se0 treatment, reaching a minimum value of 3.91 mg kg^−1^. The Zn content of the grains was reduced by 3.2% to 10.8% in MC and 13.5% to 30.1% in DY compared to under the Se0 treatment. The results of this study showed that under SOF application, only the Ca content was significantly increased in the grains from these two regions compared with under the Se0 treatment. The Fe content, however, significantly increased in the grains from DY and significantly decreased in the grains from MC. The contents of Mg, Mn, Cu, and Zn showed a significant decrease in the two areas.

### 3.3. The Content of Cd in Wheat Grains

**Figure 3:**
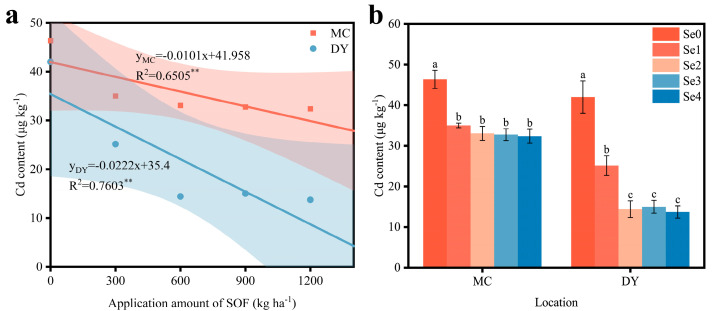
(**a**) Correlation between the application amount of selenium-containing organic fertilizers (SOF) and the cadmium (Cd) content of wheat grains in Mengcheng (MC) and Dingyuan (DY). The data are fitted into linear y = ax + b models to analyze the correlations. R^2^ is the correlation coefficient for the application amount of SOF and the Cd content. ** indicates the significant differences at 0.01 level. (**b**) Cd content of wheat grains under different ratios of SOF and organic fertilizers (OF) in MC and DY. Different lowercase letters indicate significant differences (*p* < 0.05) among different treatments using one-way ANOVA analysis with Duncan’s multiple range test and error bars show the ± standard deviation (SD). Se0: 0 kg ha^−1^ of SOF and 1200 kg ha^−1^ of OF. Se1: 300 kg ha^−1^ of SOF and 900 kg ha^−1^ of OF. Se2: 600 kg ha^−1^ of SOF and 600 kg ha^−1^ of OF. Se3: 900 kg ha^−1^ of SOF and 300 kg ha^−1^ of OF. Se4: 1200 kg ha^−1^ of SOF and 0 kg ha^−1^ of OF.

The Cd content of grains from the two regions was significantly and negatively correlated with the application amount of SOF (Figure 3a). Compared with the Se0 treatment, the reduction in Cd content in the grains from MC ranged from 24.4% to 30.1%, and in the grains from DY ranged from 40.2% to 67.3% (Figure 3b). The Cd content of the grains from MC and DY reached a minimum under the Se4 treatment, at 32.38 μg kg^−1^ and 13.73 μg kg^−1^, respectively. The findings indicated that the higher the application amount of SOF, the lower the Cd content of the grains. Additionally, the Cd content of the grains from DY had a higher decrease than that of the grains from MC.

### 3.4. The Content and Distribution of Se in Different Parts of Wheat Plants

**Figure 4:**
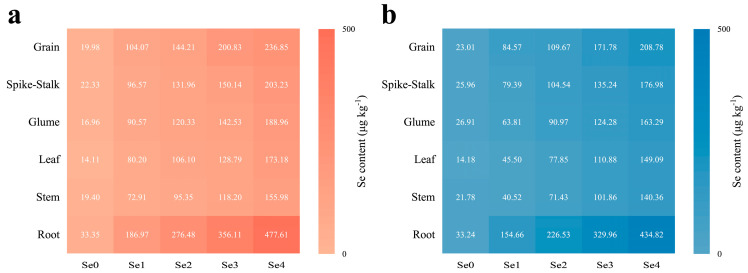
Selenium (Se) content of different parts of wheat plants (grain, spike-stalk, glume, leaf, stem, and root) under different ratios of selenium-containing organic fertilizers (SOF) and organic fertilizers (OF) in Mengcheng (**a**) and Dingyuan (**b**). Different numbers indicate the mean values of Se content in each part of wheat plants under each treatment. Se0: 0 kg ha^−1^ of SOF and 1200 kg ha^−1^ of OF. Se1: 300 kg ha^−1^ of SOF and 900 kg ha^−1^ of OF. Se2: 600 kg ha^−1^ of SOF and 600 kg ha^−1^ of OF. Se3: 900 kg ha^−1^ of SOF and 300 kg ha^−1^ of OF. Se4: 1200 kg ha^−1^ of SOF and 0 kg ha^−1^ of OF.

**Figure 5:**
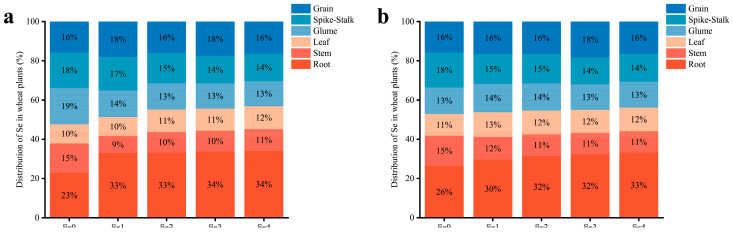
Distribution of selenium (Se) in various organs (grain, spike-stalk, glume, leaf, stem, and root) of wheat plants under different ratios of selenium-containing organic fertilizers (SOF) and organic fertilizers (OF) in Mengcheng (**a**) and Dingyuan (**b**). Se0: 0 kg ha^−1^ of SOF and 1200 kg ha^−1^ of OF. Se1: 300 kg ha^−1^ of SOF and 900 kg ha^−1^ of OF. Se2: 600 kg ha^−1^ of SOF and 600 kg ha^−1^ of OF. Se3: 900 kg ha^−1^ of SOF and 300 kg ha^−1^ of OF. Se4: 1200 kg ha^−1^ of SOF and 0 kg ha^−1^ of OF.

The application of SOF significantly enhanced the content of Se in all organs of the wheat plants in the two areas (Figure 4). The greatest increase in Se content was found in the roots. The content of Se in the roots increased 4.6 to 13.3 times in MC and 3.7 to 12.1 times in DY compared to the Se0 treatment. Furthermore, the distribution of Se in the wheat plants was altered by the application of SOF (Figure 5). The distribution of Se under the Se0 treatment in MC ranked root > glume > spike-stalk > grain > stem > leaf, whereas, in DY, it ranked root > spike-stalk > grain > stem > glume > leaf. When SOF was applied, the distribution of Se in the wheat plants in the two areas ranked as follows: root > grain > spike-stalk > glume > leaf > stem.

## 4. Discussion

The use of a variety of exogenous Se fertilizers is developing as an agricultural production technology and gradually continues to advance functional agriculture. Inorganic and organic Se fertilizers are the two primary types of Se fertilizers [31]. A study found that wheat yield varies with the method of fertilization, the period of fertilizer application, and the type of Se fertilizer. The appropriate Se concentration and application period will increase wheat production, while the opposite would prevent the growth of wheat plants [32]. Our results demonstrated that the maximum wheat yield was achieved in the two areas under study when the absolute amount of sodium selenite was 77.10 mg. However, wheat yields gradually began to decline as the application of SOF was further increased. The wheat yield reached a minimum when the absolute amount of sodium selenite was 154.20 mg. It was found that when sodium selenate was applied at 10 mg kg^−1^, the wheat yield was significantly reduced by 25–78% compared to the control treatment. This phenomenon may indicate that excessive application of Se is toxic to wheat [33]. According to some studies, Se can boost wheat yield under conditions of salinity stress and drought stress by enhancing the plant’s capability for photosynthetic activity and antioxidant defense [34,35,36,37]. However, other studies have shown that the application of Se has no significant effect on wheat yield [38,39]. It can be seen from these results that a variety of factors, including the type of Se fertilizer, application method, application period, and wheat variety, may influence the effect of Se fertilizer on wheat production.

Mineral nutrients are mineral elements and compounds that can be directly absorbed by plants from the soil environment, and different mineral nutrients perform different physiological functions for the plant [40]. For example, Ca, as a medium-level element in plants, plays an important role in maintaining the structural stability of the cell wall [41]. Zinc, as a trace element, is involved in the conversion of carbohydrates and the synthesis of proteins [42]. The application of Se fertilization might influence the mineral nutrients in crop tissues, and the degree of influence was found to vary with Se concentration, Se application method, crop variety, crop organ, etc. [43]. In this study, compared with the control treatment, with SOF application, only Ca content increased significantly in wheat grains in the two regions under study, while Fe content showed an opposite trend. A previous study found that the trend in Co content in the grains of two wheat species was not consistent after foliar and soil application of sodium selenate, which may indicate that the influence of Se on the mineral nutrients in wheat grains might differ between application methods and wheat species [44]. In this study, the content of some elements in the wheat grains showed a steady variation, which could be related to several special factors. For example, the antagonistic and synergistic effects among different ions, the role of various enzymes related to protein synthesis, and the physiological properties of wheat itself [45,46].

As a common heavy metal, Cd is one of the most serious contaminants currently harming agricultural soils. Crop roots take up and accumulate Cd from the soil, and the Cd can transfer to edible parts of the plant. Thereafter, the accumulated Cd can be transferred to people’s bodies via the food chain, posing a major threat to their health [47]. Therefore, reducing the accumulation of Cd in crops is an effective way to solve human Cd poisoning. The results of this study showed that the addition of Se significantly reduced the Cd content of wheat grains in the two regions under study. The maximum reduction was 67.3% compared to the control treatment. A previous study found that Cd content in soft wheat grains was significantly reduced by 59% to 61% when sodium selenite was applied to the soil in the range of 0.5 to 1.0 mg kg^−1^ [48]. By adding different concentrations of CdCl_2_ and sodium selenite in the wheat hydroponic experiment, Se was found to limit the accumulation of Cd in wheat plants by regulating the subcellular distribution and chemical morphology of Cd in wheat tissues and the expression of TaNramp5-a, TaNramp5-b and TaHMA_2_ in the roots [49]. Meanwhile, antagonism between Se and Cd has been found in other crops [50,51,52]. Therefore, the application of SOF in this study may have driven the wheat plants to directly inhibit the uptake of Cd at the wheat rhizosphere, thus effectively reducing the Cd content of the wheat grains.

Studies have shown that the distribution of Se in crops changed following different Se application methods [53]. In this study, we found that soil application of SOF increased the accumulation of Se in wheat roots and decreased the proportion of Se in stems and spike-stalks, resulting in the distribution of Se in wheat plants ranking as roots > grains > spike-stalks > glumes > leaves > stems. Studies have found that by applying Se ore powder to the soil, a large amount of Se was distributed in the roots of wheat, while only a small portion was present in the grains [54]. Sodium selenate and sodium selenite solutions were sprayed on the leaves at the early flowering and early filling stages, respectively. When sodium selenite was sprayed on wheat plants, the distribution trend of Se ran leaf > root > grain > glume > stem and Se was more easily transported to wheat roots. However, after spraying sodium selenate, the distribution of Se in wheat plants was found to be leaf > grain > glume > stem > root and Se was more easily transferred to the wheat grains [55]. These results may be the result of different metabolic pathways of foliar-sprayed selenate and selenite in the plants [56]. In summary, the transport and distribution of Se in wheat plants may be directly influenced by different sites and modes of Se application.

## 5. Conclusions

This study investigated the effects of selenium-containing organic fertilizer application on wheat. According to the results, wheat yields in the two areas under study significantly increased after the application of 600 kg ha^−1^ of SOF. With increased SOF application, the Ca content of the wheat grains increased and the Cd content significantly decreased. All wheat organs significantly increased in Se content following SOF application, with the greatest increase being in the roots. The distribution of Se in different organs of the wheat plant after the application of SOF ranked root > grain > spike-stalk > glume > leaf > stem. To verify the results of the current research, we will conduct repeated experiments in the same two areas in the future.

## Figures and Tables

**Table 1:** Chemical properties of surface soils (0–15 cm) in Mengcheng (MC) and Dingyuan (DY).

Property	Detection Methods	MC	DY
pH	NY/T 1377-2007	5.19	5.85
OM (g kg^−1^)	NY/T 1121.6-2006	37.2	28.7
AN (mg kg^−1^)	LY/T 1228-2015	125.8	142.3
AP (mg kg^−1^)	NY/T 1121.7-2014	23.2	8.7
AK (mg kg^−1^)	NY/T 889-2014	97.6	152.1
Total Se (mg kg^−1^)	NY/T 1104-2006	0.18	0.15
Total Cd (mg kg^−1^)	GB/T 17141-1997	0.29	0.16

OM: organic matter; AN: alkaline hydrolysis nitrogen; AP: available phosphorus; AK: available potassium; Se: selenium; Cd: cadmium.

**Table 2:** Fertilizer application ratios and absolute amount of sodium selenite of organic fertilizers (OF) and selenium-containing organic fertilizers (SOF) for each treatment in the field trial.

Variant		Treatment				
		Se0	Se1	Se2	Se3	Se4
Fertilizer application ratios (kg ha^−1^)	OF	1200	900	600	300	0
	SOF	0	300	600	900	1200
Absolute amount of sodium selenite (mg)	OF	0	0	0	0	0
	SOF	0	38.55	77.10	115.65	154.20

## Data Availability

Not applicable.
